# A miniature bio-inspired antenna for sub-6 GHz consumer wireless and biomedical diagnostic applications

**DOI:** 10.1038/s41598-026-51192-9

**Published:** 2026-05-18

**Authors:** Tapan Nahar, Sanyog Rawat, Vishal Das, Parul Pathak, Bal Virdee, Sushil Kumar Singh

**Affiliations:** 1 Information and Communication Technology, Marwadi University, Rajkot, 360003 India; 2 Electronics and Communication Engineering, Central University of Rajasthan, Ajmer, 305817 India; 3 Electronics and Communication Engineering, Manipal University Jaipur, Jaipur, 303007 India; 4 Electronics and Communication Engineering, JECRC University Jaipur, Jaipur, 303905 India; 5 Centre for Communications Technology, London Metropolitan University, London, N7 8DB UK; 6 Computer Engineering, Marwadi University, Rajkot, 360003 India

**Keywords:** Engineering, Physics

## Abstract

Miniaturization of high-performance antennas for sub-6 GHz applications such as 5G small cell, Internet of Things (IoT) devices, wearable medical system and industrial automation is essential in the near future, and the novel design methodology to realize the antenna miniaturization with high efficiency is required. In this context, this paper proposes a nature inspired antenna design using sneezewort leaf shape geometry combined with DGS (defective ground structure) to improve bandwidth and radiation features. A partial ground, the proposed antenna is very compact in size 18 × 19 × 1.6 mm^3^ (0.18λ × 0.2λ × 0.016λ) and has a wideband operation, that is, 3.16–5.42 GHz. It shows wide impedance bandwidth of 55.96% and has a highest gain of 2.1 dBi. Due to its planar, lightweight and low-profile structure, the antenna is ideal for low-cost mass production and easy integration with emerging wireless and healthcare gadgets. The multifunctional antenna lends itself to varied uses such as sub-6 GHz 5G communication, high-speed Wi-Fi, near-field vehicular radar, and industrial ISM-band devices. Besides, the antenna clearly show good prospect for many biomedical diagnostic applications, such as breast, brain, skin, lung, heart and kidney abnormalities detection, temporomandibular joint (TMJ) disorders, typhoid, bone fracture, dengue, food contaminations and lately even COVID-19. A breast tumor detection proof-of-concept is demonstrated exclusively by simulation using a breast phantom, which demonstrates the antenna sensitivity to changes in dielectric properties. These results indicate that the proposed antenna could be potential for the wireless communication in the future and also in the medical applications. Experimental validation including studies on physical phantoms and clinical studies will be included in future work.

## Introduction

Compact antennas are becoming increasingly necessary for a wide range of purposes due to the growing demand for sophisticated automated and communication equipment. Compact antennas are crucial for establishing small cells in 5G wireless networks, as fast speeds and low-latency transmission necessitate a denser architecture^1,2^. Due to size limitations and the requirement for discrete inclusion into wearable or implantable devices, these types of antennas are also essential in handheld medical apparatus^3^. Similar to this, small antennas enable smooth wireless connectivity between sensors and equipment in industrial automation settings, often in tight spaces or intricate configurations. Furthermore, to accomplish array downsizing without compromising performance, small individual components are required in the creation of high-gain antenna arrays, particularly for applications such as radar, satellite communication, and massive MIMO systems^4^.

The scarcity of small antennas that can function well at low frequencies constitutes one of the main technological obstacles, despite their importance. The primary reason for this is that the wavelength function and the dimensions of the antenna are inextricably linked; longer wavelengths at lower frequencies necessitate substantially larger antennas. One of the key areas of study is the development of antennas that can maintain strong emission quality at such frequencies while remaining electrically compact.

Antennas that integrate tiny dimensions with low-frequency functionality are becoming increasingly popular as a solution to this problem. The rising demand for wideband antennas, which must accommodate a variety of frequencies while occupying a large amount of available space, is another factor driving this trend. Consequently, efforts are being made to overcome these limitations and meet the evolving needs of contemporary technology for communication through the development of novel antenna layouts, metamaterials, and downsizing methods.

The physical size of antennas operating at sub-6 GHz frequencies—which are commonly utilized in 5G, Wi-Fi, and other wireless equipment—has been reduced using a variety of methods investigated by researchers. Increasing the dielectric constant of the substrate material is one such technique. A more compact design is made possible by using a dielectric laminate with a greater relative permittivity, which shortens the effective wavelength inside the antenna structure. However, there is a cost associated with this strategy. A larger dielectric constant tends to restrict the electromagnetic fields more tightly, which can drastically diminish the antenna’s bandwidth and radiation efficiency, even while it aids in reducing antenna size. Consequently, careful optimization is needed to strike a compromise between downsizing and respectable bandwidth, efficiency, cost, and gain performance^5–9^.

The capacity of metamaterials to regulate and modify electromagnetic energies in ways that are not achievable with ordinary substances has made them a desirable advancement in the area of small antenna design. The innovative transmission of wave properties is made possible by the unique electromagnetic properties of these intentionally manufactured materials, such as negative permittivity, negative permeability, or both. By leveraging these characteristics, metamaterials provide more design options for antennas, leading to significant improvements in radiation performance, bandwidth, and downsizing^1^. The ground surface of a 5.8 GHz antenna had been altered to incorporate a complementary split-ring resonator (CSRR) metamaterial, which altered its resonance frequency to 3.5 GHz. With an effective operating frequency range of 200 MHz, a reflection coefficient of −20.41 dB, and a gain of −3.7 dBi, this resulted in a 90% reduction ^1^.

Double- and multiband EBGs are often used for adjusting over different frequency ranges. As the dimension of a large array is reduced, inter-element coupling increases while isolation decreases in MIMO antennas. EBG constructions are effectively utilized in conjunction with antennas to increase isolation and minimize mutual coupling while preserving antenna gain and various other characteristics^10^. EBG ground is utilized in^11^ to minimize the separation among antenna components in an array by decreasing the coupling between them by 10 dB. It substantially lowered total array size while improving isolation and emission performance.

In antenna construction, a Defective Ground Structure (DGS) is a method for reducing the dimension of the antenna that can be utilized on almost any substrate^6,7,12^. DGS modifies the current distribution and electromagnetic behavior of the antenna by deliberately placing structures, such as slots, slits, or etched shapes, into the surface of the ground. These modifications may result in further inductance and capacitance, thereby reducing the resonant frequency and allowing the antenna to be physically smaller for a given operating frequency^13^.

One popular downsizing approach that enables the electrical length of the antenna to be expanded within a constrained physical space is to meander or fold the antenna structure. This method compresses the resonant structure without changing its operating frequency by bending or looping the conducting channel. It is suitable for space-constrained applications, such as mobile devices and Internet of Things systems, as it is relatively easy to deploy and allows for compact antenna designs. Higher losses, however, may result from the increased complexity of the current path, which could lower the antenna’s gain and overall radiation efficiency. Thus, rigorous optimization is necessary to strike a compromise between performance and compactness^14,15^.

A helpful method for reducing antenna size is to shorten the pins or walls, especially when aiming to lower the antenna’s height or overall profile. A virtual ground is created by introducing a conductive path between the top and bottom conducting layers, thereby reducing the resonance frequency without requiring a longer physical structure. Due to this, the design is more portable and suitable for low-profile applications, such as embedded and wearable technology. Shorting elements, however, can alter the current distribution, which may affect the radiation pattern and reduce the bandwidth. Designers must therefore carefully strike a balance between the required electromagnetic performance and size reduction^16,17^.

Loading antennas with lumped elements, such as discrete inductors or capacitors, is a practical technique for achieving size reduction by shifting the resonant frequency without increasing the antenna’s physical dimensions. This method enables compact and highly tunable antenna designs, providing precise control over the operating frequency and allowing for multiband or narrowband operation as needed. It is beneficial in reconfigurable and adaptive systems. However, integrating lumped elements adds complexity to the design and fabrication process. It may introduce additional losses due to component parasitics and interconnects, potentially degrading the overall efficiency and performance of the antenna^18^.

By placing passive resonators close to the primary radiating element, parasitic components are used in antenna design to increase impedance matching and coupling. By skillfully modifying the radiation pattern and resonant properties, this method can significantly enhance the antenna’s gain and bandwidth while also allowing for size reduction. In small and multiband designs, parasitic components are beneficial because they enhance performance without increasing the size of the primary antenna. Nevertheless, adding these components makes the design more complex overall and necessitates more space for appropriate placement, which can be challenging in situations with tight constraints^19^.

To construct antennas with electrically long current channels in a small physical space, fractal geometries utilize self-similar, repeating patterns. Fractal antennas’ special ability to facilitate multiband operation and concurrently reduce size makes them ideal for contemporary wireless devices that require the use of multiple frequency bands in a constrained area. The complex designs offer improved bandwidth characteristics and efficient space utilization. To reach their full potential, fractal designs often require sophisticated computational tools and precise manufacturing procedures due to their complexity, which presents challenges in modeling, simulation, and production^20^. Comparative analysis of size reduction techniques is presented in Table 1.

**Table 1:** Comparisons of size reduction techniques.

Technique	Principle	Advantages	Limitations
High Dielectric Constant Substrate^5^	Shortens the effective wavelength within the substrate	Reduces antenna size	Narrows bandwidth; increases dielectric loss; lowers efficiency
Meandering or Folding of Antenna Structure^14,15^	Extends electrical length in a compact space	Simple implementation; compact design	Can reduce gain; increases current path complexity
Shorting Pins or Walls^16,17^	Lowers the resonant frequency by creating a virtual ground	Reduces height/profile of the antenna	Affects radiation pattern and bandwidth
Loading with Lumped Elements^18^	Introduces inductance or capacitance to shift resonant frequency	Compact and tunable; precise frequency control	Complex integration; may introduce losses
Defective Ground Structure (DGS)^7,22^	Alters current distribution and impedance characteristics	Enhances bandwidth and efficiency; reduces size	Requires careful design; may cause unwanted radiation
Metamaterials (e.g., CSRR, CRLH) ^1^	Uses artificial materials with negative ε or μ to tailor wave behaviour	Significant size reduction; novel propagation features	Narrow bandwidth; low gain; complex fabrication
Use of Parasitic Elements^19^	Introduces passive resonators for coupling and impedance matching	Improves gain and bandwidth; enables miniaturization	Increases design complexity; requires space for placement
Fractal Geometries^23^	Uses self-similar patterns to create multiband and long path in small area	Supports multiband operation; compact design	Complex modeling and fabrication requirements
Slot or Slit Antennas^20^	Cuts in radiating surface to increase path length and alter current flow	Enhances bandwidth; enables multiband features in compact form	Potential radiation efficiency loss; needs precise manufacturing

A compact antenna not only reduces production costs but also affects other factors, such as gain, isolation, cross-polar components, grating lobes, and design complexities. There is limited space at 5G mobile stations, so a compact antenna is required for mobile stations that can work efficiently with other mobile sensors and accessories. The size of the antenna should be 0.25 times the guided wavelength to obtain moderate radiation efficiency and also reduce energy storage in near-field regions. A thicker antenna width can increase the cross-polar components. Greater PCB thickness increases the manufacturing cost in consumer electronics^3,7,21^.

Most sub-6 GHz applications require antennas with high radiation efficiencies, but common designs are not always able to provide this due to an obvious balancing act between some fundamental performance parameters. This restriction has resulted in the development of nature-based methods of designing antennas, that bring out the best in them^24,25^. Natural structures (e.g., plants, trees and flowers) can be seen as efficient electromagnetic energy harvesters, where Fibonacci-based patterns, complex geometries and radially symmetric spatial configurations play a vital role. Particularly, since the golden ratio (~ 1.618) is found everywhere in nature and the art and is related to the Fibonacci sequence, novel antennas inspired by natural entities and growth mechanisms such as leaf and branch pattern have been developed^26–28^. These natural-inspired structures, such as fractal geometries based on snowflakes^29^, trees^30^, butterfly shapes^31^, have higher perimeter to area ratio, which in turn leads to compact size, high gain and wide bandwidth antenna designs. Because frequency of antenna operation scales inversely with its physical size, these structures allow for effective miniaturization with good efficiency^24^. Different designs such as leaf^7,32^, flower^33^, spiral^26^, and butterfly-shape^34^ antennas enable better multiband characteristics and performance for WLAN and UWB applications. Advanced design techniques such as polar transformations and Gielis formulas^35,36^ allow for even more structural diversity, while variations of patch and ground planes can be used to surpass the narrowband constraints^37^. Moreover, these antennas have the capability of interference reduction with integration of notch band and frequency reconfiguration. In summary, the application of nature like fractals, golden ratio-related principles, etc. is leading to the realization of small, high performance antennas, which is expanding the range of communication systems from microwave to optical freq. ranges.

This paper^38^ demonstrates a compact bio motivated quad-port antenna for terahertz (THz) 6G high speed communication with the lotus-petal shape radiating patch and defected ground structure on polyamide substrate, which attains wideband operation (≈9–13 THz), high isolations (> 25 dB) and superior MIMO diversity performance (ECC < 0.05, DG ≈ 10, low TARC and CCL)^38^. To reduce the complexity of antenna design optimization, sophisticated nature-inspired optimization methods hybridized with multi-fidelity EM models are applied, enabling substantial computational cost reduction while preserving accuracy^39^. Along this line, several bio-inspired designs–including spider web RFID antennas for healthcare^40^, discus-shaped antennas based on the Golden Ratio for 4G^41^, and flower or fractal-shaped UWB-MIMO antennas^42^–deliver improved bandwidth, miniaturization, and radiation properties. Furthermore, two leaf-based flexible and camouflage leaf-like antennas demonstrate wideband operation with consistent gain and are compatible with contemporary wireless protocols^38^. Altogether these strategies serve to illustrate that natural inspired geometries and optimisations are particularly successful at producing miniaturised, efficient and high quality antennas suitable for future generation communication system.

Several geometrical bio-inspired antenna designs have been presented in the literature to replicate natural configurations for multiband and broadband behaviors. The results reported in^43^ till^50^ and the proposed sneezewort plant based inspired antenna clearly manifests an uniform trade-off between antenna dimension reduction, bandwidth enhancement and gain performance. The plant leaf antenna in^43^ possesses a high gain of 15.8 dB because of the relatively large electrical size (0. 95λ × 0.75λ). But its bandwidth is still narrow (8.33% and 6.59%) which indicates that while the larger apertures contribute to the improvement of the radiation efficiency, they cannot guarantee wideband operations by themselves. Also, a flower-shaped stacked array in^48^ yields the maximum gain (19.6 dB) but the array’s size, (3.81λ × 3.45λ), is too big to be adopted by compact wireless systems. These results reveal that high gain is mostly achieved through physical increase or array form. On the contrary, compact models such as the semi vine leaf antenna^44^ and leaf-shaped antenna^47^ focus on miniaturization, with antenna sizes of 0.35λ × 0.14λ and 0.24λ × 0.20λ correspondingly. Although these structures could also be operated at multiband, their gains (3.21 dB and 1. 68 dB) are greatly attenuated. This is a trade-off due to a basic principle where reduction in size causes deterioration in radiation efficiency and increase in loss. The flower-shaped monopole antenna^50^ is a more balanced performance with 72% bandwidth and the modest gain (5.25 dB), so it is more suitable for the practice. Similarly, the leaf-shaped array configuration^47^ enhances the gain to 6.56 dB over its single element counterpart, signifying that array methods can partly alleviate the low-gain constraint of miniature bio-inspired antennas. The application of metamaterial concepts (the cross-flower SRR geometry^49^) improves multiband features but does not lead to substantial gain improvement (1.5 dB). This implies that resonators help for frequency selectivity but not directly for radiation efficiency. Table 2 compares performances of bio-inspired antennas.

**Table 2:** Comparisons of performances of bio-inspired antenna.

Works	Shape	Size	Operating frequency range	Bandwidth (%)	Gain (dB)
^43^	Crops leaf	0.95λ × 0.75 λ	2.3–2.5 GHz & 4.03–4.3 GHz	8.33%, 6.59%	15.8
^44^	Semi Vine leaf	0.35λ × 0.14 λ	2.37 GHz, 3.06 GHz, 3.52 GHz, 4.28 GHz, 4.88 GHz, 6 GHz	11.97%, 4.61%, 12.43%, 6.77%, 2.46%, 11.55%	3.21
^45^	Leaf shaped patch	0.49λ × 0.49 λ	3.5–10 GHz	96.3	3.05
^46^	Spdron Fractal slot	0.56λ × 0.56 λ	2.58–5.9 GHz	78.3	4.3
^47^	Leaf shaped antenna	0.24λ × 0.20 λ	2.95–3.8 GHz	23.67%, 14.89%	1.68
^47^	Leaf shaped array	1.02λ × 1.17 λ	3.43–3.9 GHz, 4.61–4.99 GHz	13.05%, 8.08%	6.56
^48^	Flower shaped stacked array antenna	3.81 λ × 3.45 λ	5.85–7.15	20%	19.6
^49^	Cross flower geometry with split ring resonator	0.9 λ × 0.9 λ	4.01–4.82, 7.6–7.94	18.80%, 4.4%	1.5
^50^	monopole-based flower-shaped	0.51 λ × 0.51 λ	2.88 to 6.12 GHz	72%	5.25
This work	Sneezewort plant inspired antenna	0.18λ × 0.2λ × 0.016λ	3.16 GHz to 5.42 GHz	57.21%	2.1

There is an evident research gap in the design of a simple low-profile antenna structure with simultaneously high gain, wide bandwidth and small size without using complicated arrays or exotic materials. Closing this gap will be critical for future wireless applications, which will demand efficient, small, high-performance antenna systems.

The sneezewort plant, a natural occurrence, inspires the invented antenna design. The feeding structure is based on a Fibonacci pattern, and the leaf structure inspires the shape of the radiating patchwork. Design optimization has been performed to construct a structure with small dimensions, achieving an ultra-wide operational frequency range with consistent gain. This study employs a flawed ground conducting layer design in conjunction with a nature-inspired feeding method to efficiently excite the model’s four leaves, thereby overcoming the aforementioned difficulties. A practical and compact antenna suitable for contemporary wireless applications is produced by incorporating a faulty ground construction, which also enhances bandwidth performance. The proposed nature-inspired antenna is modeled with compact dimensions of 18 × 19 × 1.6 mm, which corresponds to approximately 0.18λ × 0.2λ × 0.016λ at the resonant frequency. It is designed as a single-layer structure and operates over an operating range from 3.16 to 5.42 GHz. The antenna achieves an impedance bandwidth of 57.21 MHz at the resonant frequency of 3.95 GHz, where the return loss (S11) is below −10 dB. Additionally, the antenna exhibits a highest gain of 2.1 dB, making it useful for compact and efficient wireless equipment.

There are six sections in this paper. The requirements, several approaches, and a comparative study of small antenna designs operating at sub-6 GHz frequencies are reviewed in Section I. The suggested antenna design process is described in depth in Section II. The results and a detailed analysis of the antenna’s performance are shown in Section III. The performance of the suggested antenna is compared with that of other designs documented in the literature in Section IV. Lastly, Section V highlights the antenna’s potential in biomedical diagnostics by examining its use in breast cancer detection. The study’s main conclusions and contributions are outlined in Section VI, which also highlights the usefulness of the proposed antenna design and its potential applications.

## Antenna design

Natural plant and tree forms are extensively exploited as bio-inspired models in microwave and antenna design, as they have naturally evolved geometries that maximize the harvesting of electromagnetic energy. Vegetation positions its leaves and stems so that incident sunlight (similar to EM waves) is intercepted efficiently for photosynthesis. This concept drives natural antenna shapes, which have consistently proven to offer good bandwidth, multiband characteristics, and compactness. As a general design principle, bio-inspired antennas are usually constituted by four stages: (i) analyzing and understanding the natural growth rule or geometric pattern in nature, (ii) modeling this rule or pattern into an abstract mathematical version that can be scaled up and down, (iii) mapping the abstract model into metallic/planar geometry considering impedance matching, radiation efficiency, and manufacturability, and (iv) fabricating the antenna and testing its performance.

**Fig. 1:**
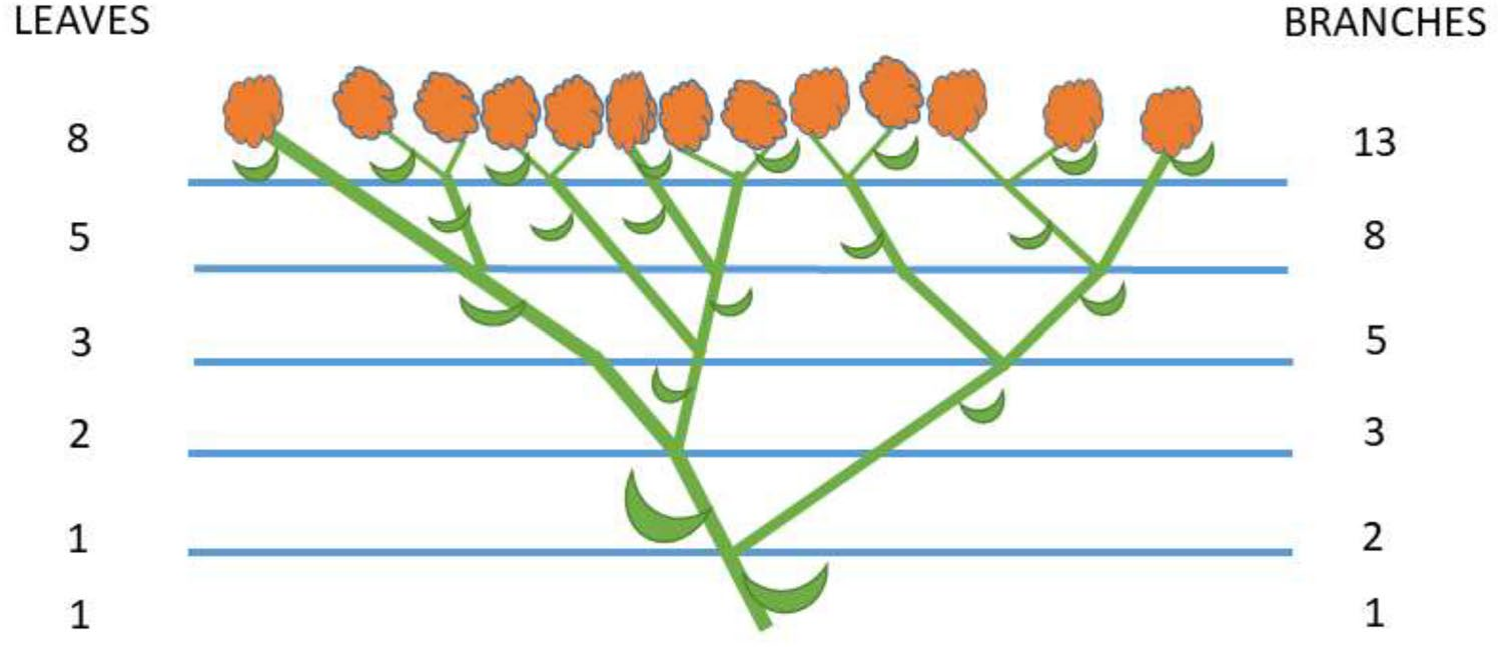
Structure of sneezewort plant^52^.

In this study, the antenna curve is derived from the golden ratio and the Fibonacci series, as previously demonstrated in^28^. The Golden Ratio is evident in nature and classical design—in Greek architecture, art, and urns, as well as on playing cards and in the paintings of Leonardo da Vinci. The Fibonacci sequence drives the branching, where each element represents the sum of the two previous elements in the sequence. A well-known instance is the sneezewort (Achillea ptarmica), as seen in Fig. 1. If we take the number of branches at stages 1 and 2 to be 1 and 2, respectively, then the number at stage 3 becomes 1 + 2 = 3 as per the recurrence relation^12,28,51^.

1 $$F_{n} = F_{n - 1} + F_{n - 2}$$

The same holds for the leaf arrangement, where F0 = F1 = 1, F2 = 2, F3 = 3, and so on. As shown in Fig. 1, every level is the sum of the previous two levels. A significant design rule in this antenna is the use of the golden ratio to calculate the widths of successive branches. If the first width is W1, then the widths that follow are:

$$W_{2} = \, W_{1} / \, 1.618; \, W_{3} = \, W_{1} - W_{2}$$

This is carried out recursively for all higher-order branches to maintain geometric proportionality and equal current distribution. Moreover, the branching angle follows the golden angle of 137.5°, which is the angle that minimizes mutual shadows in plants. The angle between two successive branches is (180–137.5)°, which ensures the uniform spreading and the reduction of coupling (important to maintain balanced radiation patterns in the antenna array structure)^12^.

The sneezewort plant was used as a model because it lies at the intersection of geometric simplicity, clear Fibonacci-based ordering, and structural symmetry, and can be readily converted into engineering patterns. Its shape allows for easy mathematical modeling, compact and fabrication-friendly designs, as well as feature spacing that can be utilized in miniaturized broadband electromagnetic structures. In comparison to other Fibonacci-based botanical sources, which are generally combined with curves, fills, or inappropriate 3D forms, the sneezewort offers a more pragmatic, scalable, and electromagnetically sound template for bio-inspired antenna development.

Building antenna array excitation networks is challenging since losses worsen at every tier, as the impedance does not match well. The splitting of the sneezewort tree, as seen in Fig. 1, gave rise to the excitation network. The sneezewort-type splitting is employed in the excitation network because its hierarchical and self-similar branching structure offers a natural template for realizing a controlled and progressively diminished power division at the terminal radiating elements. In this microstrip network representation of the biological splitting scheme, each branching stage corresponds to a proportional reduction in the width of the arms, with a factor derived from the golden ratio, thereby ensuring impedance continuity and minimizing mismatch loss. No leaf-like flanges can be added in the intermediate stages, as these protrusions, once made in metal, work as parasitic radiating stubs which excite spurious modes, radiate more with fringing fields, and generate undesired resonances—all this leads to an unbalanced excitation. Only at the final stage are leaf-shaped elements used, where radiation is deliberately allowed.

**Fig. 2:**
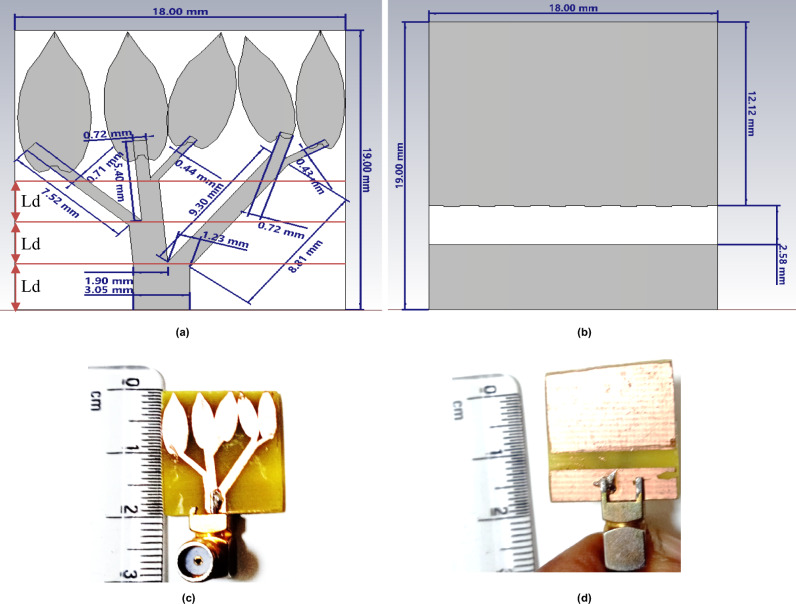
Geometry of sneezewort plant inspired antenna (**a**) top structure (**b**) bottom structure (**c**) top of fabricated antenna (**d**) bottom of fabricated antenna.

The initial branch width for branching is W_1_ (3.05 mm), which corresponds to a 50 Ohm microstrip line width for a FR4 substrate with a relative permittivity of 4.4, loss tangent of 0.025, and height of 1.6 mm. In the final stage, the leaf shapes are broadly similar, except for a few thinner leaves in the center, which serve to maintain separation. Since the FR4 substrate is readily accessible at a low cost, it was chosen. New arm widths are calculated by dividing the main stem width by the golden ratio and then subtracting the main arm and first arm widths (W_2_ = 3.05/1.618 = 1.9 and W_3_ = 3.05–1.9 = 1.14). A similar concept is used at higher levels. The branching element lengths are organized, as depicted in Fig. 2, so that the vertical length (Ld) of each stage is the same. Ld is chosen to be 3 mm, after optimization with a slot in the ground plane, which is about 0.04 of the resonant wavelength. For narrowband design with a full ground plane the feed line length is generally kept as half wavelength; however, for wideband performance using defected ground plane the feed length can be varied in a certain range. The DGS also allows for the customization of multiple resonant modes within a wide range of frequencies. Besides, a last stage optimization is made to reduce the overlapping of the leaf element. A leaf-shaped patch is firstly generated by the polar equation. r = 2a (1 + cosθ)^47,53^. This formula is given in polar coordinates, and the Cartesian coordinates can be calculated as follows: x = rcosθ and y = rsinθ^7,53^. As θ ranges from 0 to 360° and setting r = 6 mm, a cardioid is obtained. The apex of this pattern is then extended to 9.6 mm (about 0.125 of the operating wavelength) for a leaf-like geometry. Further refinement of the leaf size, length and width as well as by introducing a slot in the ground place, is performed to reduce overlap between leafs and to satisfy specified radiation characteristics.

A slot was excavated from the ground to provide wideband characteristics in the Sub-6 GHz region. The top and bottom sides of the simulated sneezewort-inspired antenna are shown in Fig. 2a, and b and fabricated prototype is shown in Fig. 2c and d. The novelty of the antenna lies in combining a sneezewort-based branching method with a width-scaling approach based on the golden ratio to form a balanced and impedance-matched excitation network, rather than relying solely on Fibonacci patterns for the design of the radiating element, as seen in previous works. The novel bio-inspired feed mechanism, combined with a ground-plane slot, enables wideband Sub-6-GHz operation on an inexpensive FR4 substrate, resulting in an overall compact and easy-to-fabricate configuration that has not been reported in the literature of nature-inspired antennas.

## Results and discussions

**Fig. 3:**
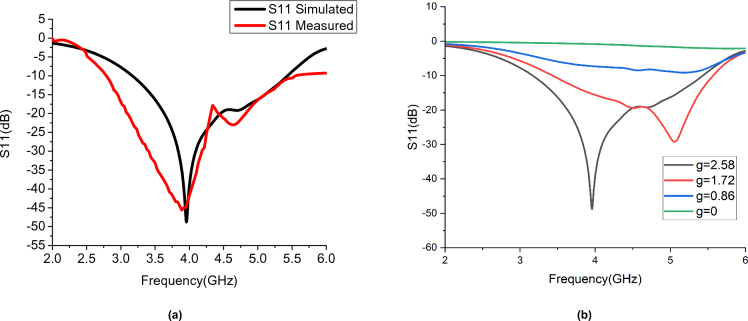
(**a**) Observed and modeled S11 vs. freq. trace of sneezewort plant inspired antenna (**b**) S11 vs. freq. trace for various gap width (g).

The Fig. 3a depicts the observed and modeled S11 vs. frequency trace of the suggested sneezewort plant-inspired antenna. The simulated antenna resonates between 3.16 and 5.41 GHz, offering a bandwidth of 56.96% at 3.95 GHz. At the resonance freq., S11 is -48 dB, indicating excellent impedance matching and low return loss. The fabricated antenna is tuned from 2.8 to 5.5 GHz, achieving a minimum S11 of −46.5 dB at 3.9 GHz.

Figure 3b depicts the influence of varying gap widths in the ground plane on the S11 vs. frequency trace. If there is no gap in the ground, it can be proven that the proposed antenna does not resonate in the lower frequency region. The resonance might be changed to higher frequencies. The resonance is pushed to higher frequencies, and the bandwidth is enhanced with a gap width of 1.72 mm. The suggested antenna offers maximum bandwidth and acceptable impedance matching within the necessary operating spectrum, with a gap width of 2.58 mm.

**Fig. 4:**
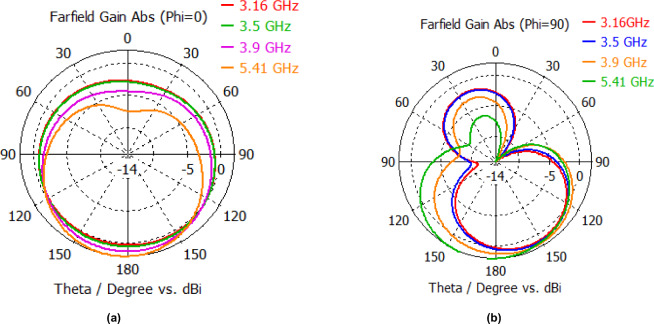
Two dimensional radiation characteristics at multiple frequencies (**a**) Phi = 0 (**b**) Phi = 90.

Figure 4a and b illustrate the two-dimensional radiation patterns at different operating frequencies for Phi = 0° and Phi = 90°, respectively. The main lobe at higher frequencies is more directional, resulting in a higher gain. A significant decrease in the side-lobe level and back radiation is also observed as the frequency increases, indicating improved radiation performance. Despite these changes, the main lobe orientation remains invariant within the entire frequency band, indicating that the directional behavior of the antenna was not affected by frequency.

**Fig. 5:**
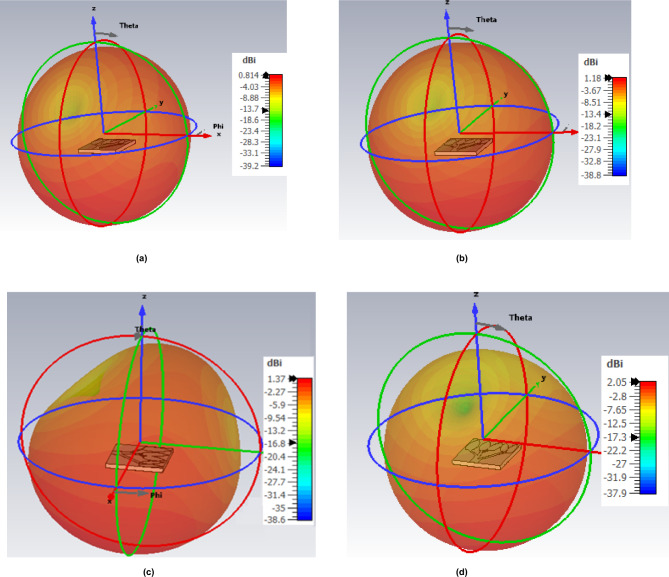
Three dimensional radiation characteristics at (**a**) 3.16 GHz (**b**) 3.5 GHz (**c**) 3.9 GHz (**d**) 5.47 GHz.

Figure 5a–d shows the three-dimensional radiation patterns of the proposed antenna at 3.16 GHz, 3.5 GHz, 3.9 GHz, and 5.47 GHz. The peak gain exhibits a fluctuation of less than 3 dB within this frequency band, indicating stable radiation performance over the antenna’s operating bandwidth. Furthermore, the direction of the primary radiation lobes remains unchanged, implying that the beam orientation is independent of frequency. The corresponding peak gains are 0.814 dB, 1.18 dB, 1.37 dB, and 2.05 dB at 3.16 GHz, 3.5 GHz, 3.9 GHz, and 5.47 GHz, respectively.

**Fig. 6:**
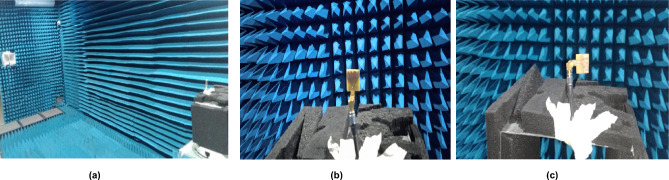
(**a**) Measurement setup in Anechoic chamber (**b**) Antenna setup for E-pattern measurement (**c**) Antenna setup for H –pattern measurement.

Using an Agilent VNA, the S₁₁ parameter of the fabricated antenna was measured at the Government Women’s Engineering College, Ajmer. The radiation patterns were measured in the anechoic chamber of our institute by employing the fabricated antenna as the receiving antenna. The test antenna was connected to an RF receiver (spectrum analyzer) and mounted on a motorized turntable. The standard horn antenna (gain ≈ 10 dB) was employed as the transmitting antenna, driven by an RF generator during pattern measurement. The measurement setup is shown in the Fig. 6a. The horn-to-AUT distance was maintained at approximately 5 m to satisfy the far-field condition. Both antennas are connected to the VNA at one end through RF cables to collect data, and a PC at the other end to store the far-field measurement results. The sweep generator supplied the necessary test frequencies, and the turntable rotated the AUT through 0–360° to obtain the entire radiation characteristics. Antenna alignment for the E-plane and H-plane is shown in Fig. 6b and c. Co-polarized measurements correspond to both the transmit and receive antennas having the same polarization. In contrast, the cross-polarization measurements are performed by rotating the transmit antenna alone by 90° with the receive antenna fixed.

**Fig. 7:**
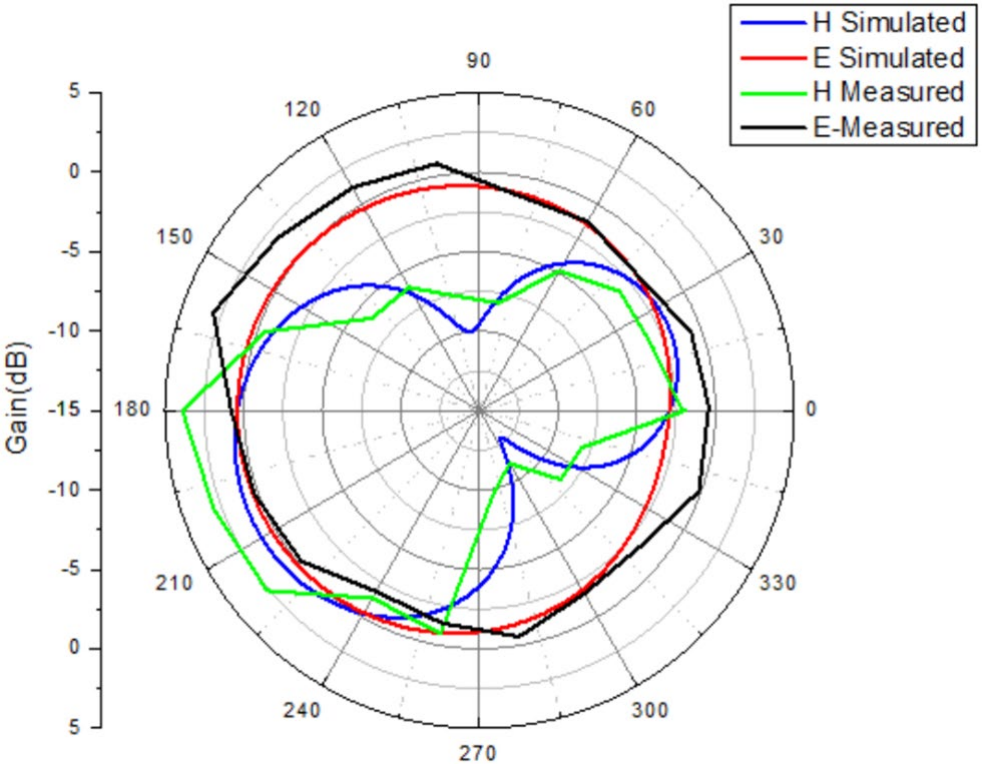
Simulated and measured radiation characteristics of sneezewort plant inspired antenna at 3.9 GHz.

Figure 7 depicts the observed E-field radiation characteristics, the H-field radiation characteristics, and the modeled E and H-field radiation characteristics at 3.9 GHz. It can be noted that the maximum gain of the E-field and H-field patterns closely matches their modeled versions; however, the shape of the observed radiation characteristics varies somewhat due to manufacturing imperfections and solder joints. The gain degradation observed, from 2.1 dBi in simulation to 1.338 dBi in the fabricated prototype, is primarily due to substrate losses, including a higher-than-expected dielectric loss tangent and milling material inhomogeneities. Other sources of error include minor soldering imperfections, connector mismatches, and finite precision in the etching of the conducting layer, which can result in slight variations in the local impedance and parasitic currents. The combined effect of these factors is to reduce the realized gain, and measurement tolerances may further contribute to this reduction.

**Fig. 8:**
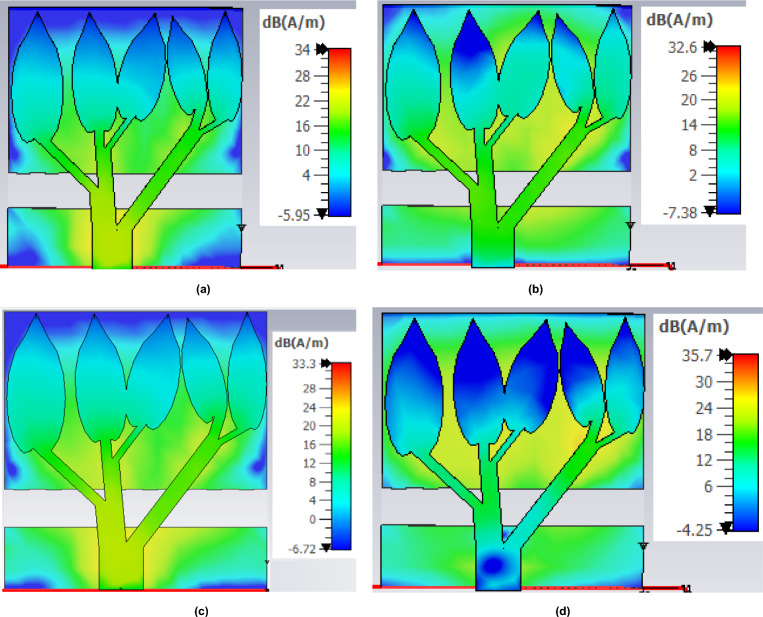
Surface current distribution of nature inspired antenna at (**a**) 3.16 GHz (**b**) 3.5 GHz (**c**) 3.95 GHz (**d**) 5.47 GHz.

Figure 8a, b, c, and d plots the simulated surface currents of the proposed antenna at 3.16 GHz, 3.5 GHz, 3.9 GHz, and 5.47 GHz, respectively. It can be observed that the current is densest in the central stem and the radiating branches, which actively participate in antenna radiation. Stronger current distributions in the upper sections have been marked with the green-to-yellow part, as the blue background indicates a low current level when the current intensity is decreasing off the main radiating routes. This pattern also indicates that the antenna is well excited on the radiating surfaces, and there are no visible unwanted currents collected around the ground plane. In general, the surface current verifies the validity of the designed current flow model, and the result ensures the stable radiation performance of the proposed antenna within the simulated frequency band. The top leaf-shaped structures have high current densities with values of 34 dB (A/m), 32.6 dB(A/m), 33.3 dB(A/m), and 35.7 dB(A/m) at 3.16 GHz, 3.5 GHz, 3.9 GHz, and 5.47 GHz, respectively.

**Fig. 9:**
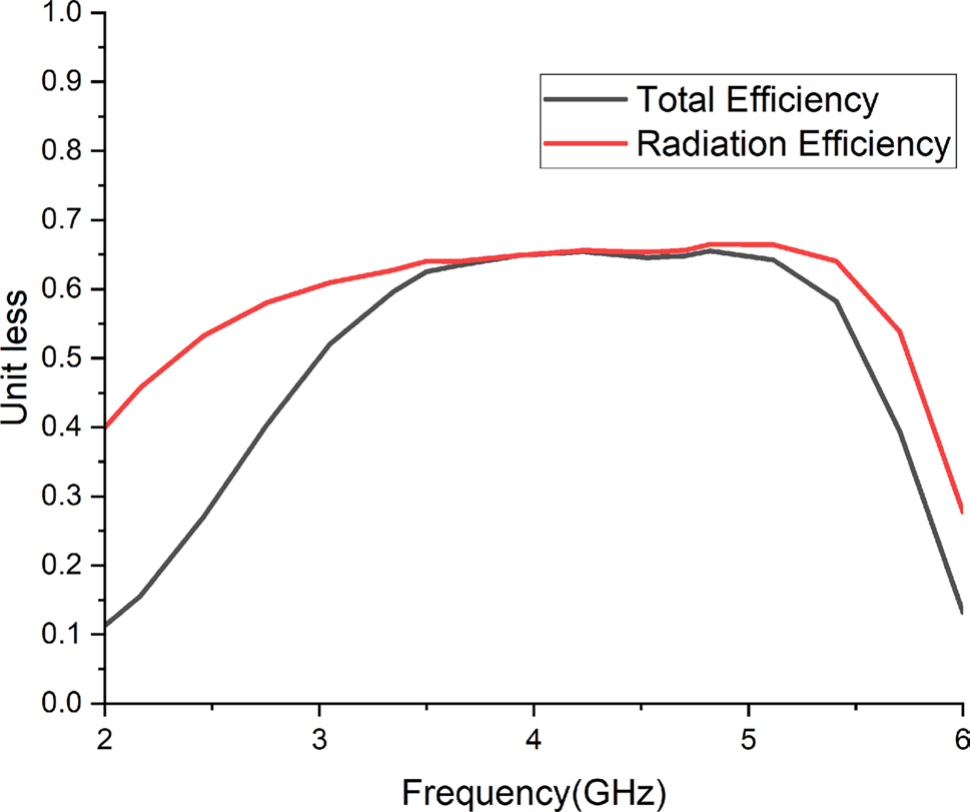
Efficiency vs. freq. trace of proposed nature inspired antenna.

Figure 9 illustrates the relationship between total efficiency, radiation efficiency, and frequency. Within the working spectrum, both efficiencies exceed 60%. The most significant radiation efficiency and total efficiency are 66.9% and 65.6%, respectively.

**Fig. 10:**
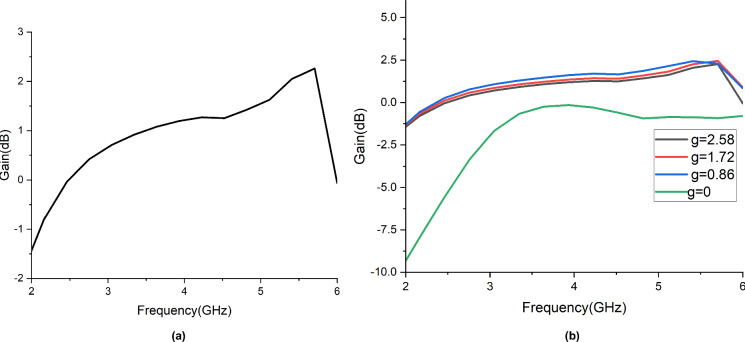
(**a**) Gain vs. freq. trace of nature inspired antenna (**b**) Gain vs. freq. trace for various gap width.

Figure 10a depicts a gain vs. freq. trace of a nature-inspired antenna. At 5.47 GHz, maximum gain is 2.09 dB—the gain changes by less than 3 dB across the operational bands. Figure 10b depicts the effect of different gap widths on gain. It should be noted that in the absence of a slot in the ground, the resonance shifts to a higher spectrum, reducing gain at the desired band. Gain is improved for gap widths of 0.86 mm and 1.72 mm because the reflector area is increased. Compared to other gap widths, the gain is reduced, but the bandwidth is improved for a gap width of 2.58 mm.

The comparison of the simulated and measured performance of the antenna shows good agreement, which confirms the proposed design. The simulated antenna yields 3.16–5.42 GHz operating band with impedance bandwidth of 57.21% at 3.95 GHz and 2.1 dBi of maximum gain. By contrast, the measured results demonstrate a slightly broader band in which the antenna works at 2.8–5.5 GHz with an impedance bandwidth of 69.23% at3.9 GHz, and the peak gain is fallen down to 1.338 dBi. A variation of 1.3% in resonant frequency, a 20% increase in bandwidth and a 0.76 dB (≈36%) reduction in gain are found. The small frequency deviation signifies good impedance stability, and the increasing bandwidth in the measured results is mainly attributed to additional losses and parasitic effects caused by fabrication tolerance and the DGS. The decrease of gain is mainly caused by the substrate loss, connector mismatching and soldering defects at the feeding point as well as the measurement error. In summary, these differences are acceptable when implementing practical antenna and well demonstrate the feasibility and validity of the proposed design.

### Performance comparison of the proposed nature-inspired antenna to the reported 5G microwave antenna

Table 3 compares the outcomes of the suggested antenna inspired by nature with those of the reported 5G microwave antenna. Notably, the designed antenna has dimensions of 0.18λ × 0.2 λ × 0.016λ, which are small in comparison to the antenna’s maximum working wavelength (λ) and previously reported antennas^54–60^. The recommended modeled antenna has a maximum gain of 2.1 dB and a bandwidth of 57.21 percent. The manufactured antenna has a maximum bandwidth of 69.23% and a maximum gain of 1.338 dB. The suggested antenna has a wider freq. range than the current antennas^54,55,58–60^. Azim et al.^56^ has a broader bandwidth than the proposed antenna, but it has a lower gain. The peak gain of the recommended antenna is more than that of existing antennas^55–57^ but less than that of^54,58–60^, while its dimension is smaller than that of^54–60^ and its bandwidth is greater than that of^54,58–60^. Due to its compact dimensions, wide bandwidth, reasonable gain, low profile, low cost, and simplicity of manufacture, the nature-inspired antenna is well-suited for sub-6 GHz 5 G applications.

**Table 3:** Comparisons of projected nature-inspired antenna parameters to existing sub-6 GHz antennas.

Work	Dimension	Freq. range (GHz)	Impedance bandwidth (S11 < 10)	Max gain (dB)
Proposed (Modeled)	18 × 19 × 1.6 (0.18λ × 0.2 λ × 0.016λ)	3.16 to 5.42	57.21 (3.95 GHz)	2.1
Proposed (Observed)	18 × 19 × 1.6 (0.18λ × 0.2 λ × 0.016λ)	2.8 to 5.5	69.23 (3.9 GHz)	1.338
^54^	50 × 50 (0.51λ × 0.51 λ)	3.1 to 3.8 and 5.1 to 6.19	20 (3.5 GHz)	2.85
^55^	30 × 29 × 9 (0.238λ × 0.23 λ × 0.07 λ)	2.38 to 2.77	16.25 (2.4 GHz)	1.42
^56^	20 × 28 × 1.6 (0.2λ × 0.28 λ × 0.016 λ)	3.05 to 5.82	79.14 (3.5 GHz)	0.34
^57^	45 × 64 (0.36λ × 0.52 λ)	2.45 and 5.8	4.5 3.8	− 1.8 1.1
^58^	40 × 40 × 1.124 (0.33λ × 0.33 λ × 0.009 λ)	2.36 to 2.74 and 5.14 to 6.18	15.57 (2.4 GHz) 18.90 (5.5 GHz)	0.74 2.3
^59^	30 × 34 × 0.8 (0.19λ × 0.21 λ × 0.005λ)	1.9 to 2.17 and 3.4 to 3.6 and 5.15 to 5.35	13.5 (2 GHz) 5.71 (3.5 GHz) 4 (5 GHz)	− 1 1 3.5
^60^	24 × 20 (0.19λ × 0.156 λ)	2.35 to 2.47and 5.08 to 5.36 and 8.01 to 8.31	5 (2.4 GHz) 5.3 (5.26 GHz) 3.66 (8.18 GHz)	1.2 to 3.18

### Applications of the proposed nature-inspired antenna

The antenna’s compact dimensions (0.18λ × 0.2λ × 0.016λ), wide impedance bandwidth of 55.96%, consistent peak gain of 2.1 dBi, low gain fluctuation (1.91 dB), and high efficiency (65.6%) make it ideal for a variety of wireless and healthcare applications. Its flat, lightweight, and low-profile design allows for simple integration into devices and systems while also being cost-effective to manufacture. The antenna operates in the 3.16–5.42 GHz spectrum, which includes key sub-6 GHz frequencies utilized in 4G/5G mobile communication, Wi-Fi, IoT networks, short-range radar, wireless power transmission, and industrial ISM-band sensing.

This frequency range is handy in biomedical applications due to its ability to penetrate soft tissues and provide non-invasive diagnostic capabilities. The antenna’s performance characteristics make it ideal for advanced healthcare technologies, including breast cancer detection, skin cancer screening, brain tumor identification, monitoring the impact of COVID-19 and dengue viruses, lung infection detection, knee ligament evaluation, bone fracture diagnosis, and general anomaly detection via microwave imaging. Its broadband response, steady radiation properties, and high sensitivity to dielectric changes enable the precise identification of physiological irregularities in real-time, making it a crucial component in the development of biomedical diagnostic systems.

One of the strong practical applications for the proposed antenna is breast cancer detection in a microwave imaging system. In this paper the illustrative example is a simulation based concept under ideal circumstances, showing that the antenna can be developed into a small and power efficient sensing element. The results reveal the ability of the antenna to differentiate a 0.1 mm tumor in a model of breast phantom, which clearly denotes the sensitivity of the antenna. But in real situations, it is anticipated that initially tumor dimensions detectable are in the range of millimeter, considered suitable for early cancer diagnosis, as can be seen from simulated noises and tissue heterogeneity. Hence, the results although demonstrate the concept and hold significant promise for biomedical imaging applications, experimental validation is considered as future work.

**Fig. 11:**
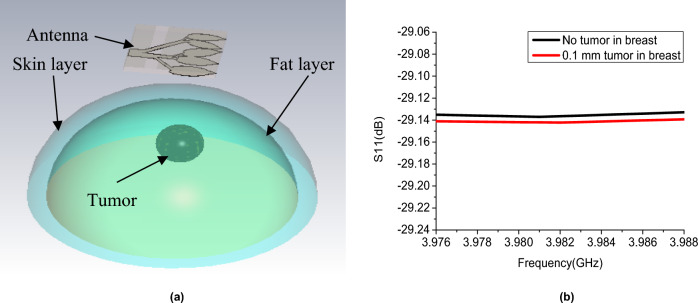
(**a**) Antenna with breast phantom (**b**) S11 versus frequency plot for breast with tumor and without tumor.

A 3-D voxel-based breast phantom with a homogeneous skin layer and fat layer was simulated in CST (Computer Simulation Technology) Microwave Studio, and the dielectric properties were taken from Table 3^61–63^. Figure 11a presents the breast phantom with a tumor and antenna. Based on the dielectric constant and loss values for breast tissue from the literature and employed in CST simulations, the layers of the breast phantom were assigned material properties with tissue data experimentally validated. The skin layer was simulated with a relative permittivity of 36.7, an electrical conductivity of 2.34 S/m, a thickness of 4 mm, and a density of 1109 kg/m^3^. The fat beneath it had a relative permittivity of 4.84, conductivity of 0.262 S/m, a thickness of 26 mm, and a density of 911 kg/m^3^. The malignant tumor inclusion was also simulated with a significantly larger dielectric contrast, characterized by a relative permittivity of 54.9, an electrical conductivity of 4 S/m, and a density of 1058 kg/m^3^. These significant differences in dielectric responses of normal and malignant tissues are also the underlying reasons for measurable differences in electromagnetic scattering and reflection (S11), from which the suggested antenna system can detect the tiny anomalies in the breast phantom.

The proposed antenna was positioned 25 mm above the hemispherical phantom, with its center directed towards the phantom to illuminate it efficiently and minimize direct electromagnetic coupling. A transient solver with an open boundary was employed to achieve broadband operation in the frequency band of 3.16–5.47 GHz, and local mesh refinement was implemented in the tumor and tissue boundary regions to enhance the accuracy of field resolution. The malignant inclusion was modeled as a spherical tumor with a radius of 0.1 mm within the fat space. The associated disturbance of the dielectric resulted in a detectable change in the reflection coefficient, demonstrating the antenna’s sensitivity to minor variations in the dielectric. With a comparison of the most recent microwave imaging works on minimum detectable tumor size, which have been shown in the range of 2–20 mm^64–68^, the detection of a 0.1-mm anomaly in this work endows a great deal more sensitivity, thus it further proves the proposed antenna’s effectiveness and prospect for early-stage breast cancer detection. The antenna uses the scattered signal from the breast as a parameter to determine whether a tumor is present and to determine its size and location^61,69^. In the presence of a tumor in the breast, the dielectric parameters of the breast are modified, which modifies the wave transmission characteristics (reflection, diffraction, and scattering) and emission characteristics. According to variation in the dielectric properties, the specific absorption rate and reflection coefficient (S11) will be varied^62,70^. By establishing an empirical relationship between the antenna’s reflection coefficient (S11) and the presence of a tumor, it becomes possible not only to detect the existence of a breast tumor but also to estimate its size within a breast phantom model. For validation, a spherical tumor with a diameter of 0.1 mm—characterized by specific electrical properties as detailed in Table 4—was embedded within a tissue-equivalent breast phantom. The reflection coefficient (S11) was measured under two conditions: with and without the tumor. As illustrated in Fig. 11b, the S11 value in the presence of the tumor was recorded as −29.14446 dB, whereas it measured −29.13619 dB in the absence of the tumor. Though the numerical difference is slight, the detectable variation in S11 signifies a change in the dielectric environment, thereby indicating the presence of the tumor. This sensitivity to dielectric contrast highlights the antenna’s potential as a non-invasive sensor for early-stage breast cancer detection.

**Table 4:** Dielectric characteristics of Breast phantom layers and tumor^61–63^.

Tissue	Electrical permittivity (ε_r_)(F/m)	Electrical conductance (S/m)	Density (Kg/m^3^) (Ρ)	Thickness (mm)
Skin layer	36.7	2.34	1109	4
Fat	4.84	0.262	911	26
Tumor(malignant)	54.9	4	1058	–

Detecting a 0.1 mm tumor in the breast phantom study is a highly idealized scenario to highlight the high sensitivity of the antenna. Real breast tissue is heterogeneous, and the dielectric contrasts of small tumors are not as exaggerated; therefore, detection at the sub-millimeter size may not be achievable. In vivo realistic detection is usually limited to tumors of a few millimeters; thus, the phantom result is used mainly as a proof-of-concept rather than representing the limits of clinical resolution.

**Fig. 12:**
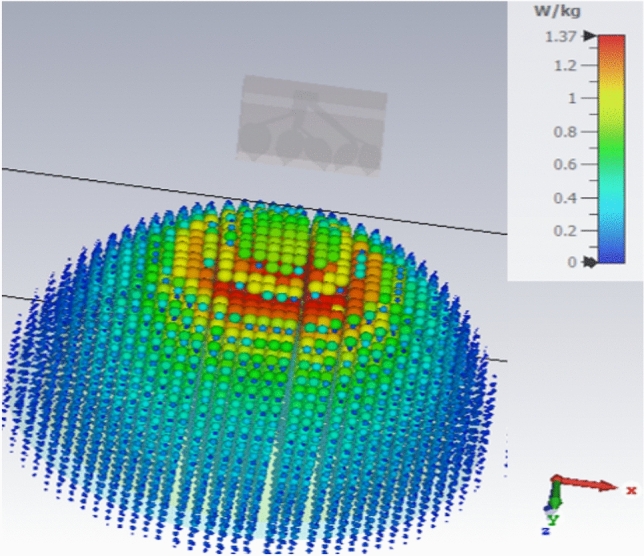
SAR of Antenna by placing over breast phantom.

The antenna was located at a standoff distance of 25 mm from the multi-layer tissue phantom to assess the electromagnetic exposure in CST Microwave Studio. From SAR analysis, the peak spatial-averaged SAR value over 1 g was found to be 1.37212 W/kg, which is significantly lower than the regulatory limit of 1.6 W/kg as per Indian guidelines, as shown in Fig. 12. Therefore, the suggested design meets national safety standards, demonstrating its applicability to real-time biomedical or communication applications near humans.

Table 5 provides a concise summary of the influence of different breast tumor diameters on SAR, S11, and resonant frequency under a microwave-based detection antenna model. As the tumor size decreases from 10 to 0.1 mm, the SAR value computed for 1 g increases for the tissue sample from 1.31456 to 1.37212 W/kg. This increase is associated with smaller tumors, which have higher dielectric constants than normal breast tissue and thus produce a stronger localized electromagnetic field concentration, while absorbing slightly more energy. In addition, as the tumor size decreases, the reflection coefficient (S11) tends to become more negative, from −28.06 to −29.14 dB, indicating better impedance matching of the antenna and higher sensitivity for small dielectric perturbations induced by the tumor. This result indicates that even a tiny tumor can alter the electromagnetic environment sufficiently to cause a detectable decrease in reflected power. Furthermore, small but steady shifts in the resonant frequency are observed in the interval from 4.025 to 4.029 GHz, representing the variation in the effective permittivity due to different tumor sizes. In summary, the gradual variation of SAR, S11, and frequency indicates that the proposed antenna can detect small tumors, which implies great potential applicability in the detection of early-stage breast cancer.

**Table 5:** Observed variations in antenna parameters as per different tumor radiuses.

Tumor radius	SAR(W/Kg) for 1 gm	S11(dB)	Frequency (GHz)
10 mm	1.31456	-28.06	4.025035
5 mm	1.36174	-28.65	4.035452
2.5 mm	1.36994	-28.87	4.034383
0.1 mm	1.37212	-29.14	4.029317

Table 6 presents a comparison of the proposed system with currently available microwave imaging techniques in the literature. It is observed that, among the presented designs, the proposed antenna has the smallest size of 0.18λ × 0.2λ while achieving optimal S11 and SAR parameters simultaneously. Importantly, it is capable of detecting tumors as small as 0.1 mm in radius, which is better than that of all the cited works. The difference is that the 0.82λ × 0.82λ large patch antenna in^64^ is designed solely based on S11, resulting in a minimum detectable tumor radius of 2 mm. Additionally, the S11 & SAR method in^66^ utilizes a 0.95λ × 0.91λ antenna with a minimum detection accuracy of 3 mm. The technique in^67^, which involves S11, S12, and E-field parameters, however, requires an antenna size of 0.23λ × 0.23λ to detect tumors of at least 5 mm in diameter. Finally, a 0.38λ × 0.2λ antenna is employed for the S21 measurements in the configuration described in^65^, and tumors of size equal to 10 mm are identified.

**Table 6:** Comparison with existing works.

Works	Antenna size	Detection parameters	Minimum tumor radius (mm)
This work	0.18λ × 0.2 λ	S11, SAR	0.1
^64^	0.82λ × 0.82 λ	S11	2
^66^	0.95λ × 0.91 λ	S11, SAR	3
^67^	0.23λ × 0.23 λ	S11,S12,E field	5
^65^	0.38 λ × 0.2 λ	S21	10

The proposed antenna has not been tested for its robustness under temperature and humidity variations in this work. Nevertheless, considering that a conventional FR4 substrate is utilized, the antenna should be moderately sensitive to the surrounding environment. Changes in temperature and humidity could also affect the dielectric constant and loss tangent of the substrate, resulting in minor shifts in resonant frequency, impedance matching, and radiation efficiency. Under normal circumstances, these effects are small and the performance of most consumer and indoor products is not noticeably degraded. Nevertheless, environmental extremes may cause more significant changes. A comprehensive environmental reliability study including temperature and humidity controlled testing is reserved for future work to completely evaluate the robustness of the proposed antenna design. In terms of the potential for biomedical application, the present study provides a simulation-based proof-of-concept under idealized conditions showing that the antenna is sensitive to dielectric differences. However, an in-depth uncertainty analysis and sensitivity analysis is to be explored and it is part of our future work. In practice, detection accuracy may be affected by tissue heterogeneity, measurement noise, modeling assumption, environmental variation and so on. Hence, the proposed methodology proves to be robust and it is expected that a full uncertainty quantification, sensitivity analysis, and experimental validation with physical phantoms and under realistic conditions will be performed in future work to further establish the reliability and clinical feasibility of the approach.

## Conclusion

In this paper, a compact bio-inspired antenna for sub-6 GHz applications, mainly in the 3.16–5.42 GHz frequency range, is proposed. The electrical size of the proposed antenna is 0.18λ × 0.2λ × 0.016λ and it has a bandwidth of 55.96%, a gain of 2.1 dBi and an efficiency of 65.6% which can be used in wireless communication, radar system and biomedical imaging. Its planar, thin, and low-profile design enables low-cost mass production and effortless integration, meanwhile the defected ground structure (DGS) and bio-inspired shape are used to improve the bandwidth and the whole performance. A breast cancer detection example is shown as a simulation-based proof of concept, the capability of the antenna in sensing alterations of tissue dielectric. While performed in ideal media, this underscores its prospective use in wider biomedical fields like brain, lung, skin, and bone applications. As a whole, the multi-functional feature of the proposed antenna renders it a promising candidate for the future healthcare system and the next generation of wireless communication technology, with experimental biomedical validation as future work.

## Data Availability

The datasets used and/or analysed during the current study available from the corresponding author on reasonable request.
